# Peripheral Spexin Inhibited Food Intake in Mice

**DOI:** 10.1155/2020/4913785

**Published:** 2020-08-05

**Authors:** Shuangyu Lv, Yuchen Zhou, Yu Feng, Xiaomei Zhang, Xinyue Wang, Yanjie Yang, Xinchun Wang

**Affiliations:** ^1^Institute of Molecular Medicine, School of Basic Medical Sciences, Henan University, Kaifeng 475004, China; ^2^The First Affiliated Hospital of Henan University, Kaifeng 475001, China

## Abstract

Spexin (SPX, NPQ), a novel endogenous neuropeptide, was firstly identified by bioinformatics. Spexin gene and protein widely distributed in the central nervous system and peripheral tissues, such as the hypothalamus and digestive tract. The role of spexin in appetite regulation in mammalian is still unclear. The present study was designed to investigate the mechanism and effect of peripheral spexin on food intake in mice. During the light period, an intraperitoneal (i.p.) injection of spexin (10 nmol/mouse) significantly inhibited cumulative food intake at 2, 4, and 6 h after treatment in fasted mice. During the dark period, spexin (1 and 10 nmol/mouse, i.p.) significantly suppressed cumulative food intake at 4 and 6 h after treatment in freely feeding mice. The GALR3 antagonist SNAP37889, not GALR2 antagonist, significantly antagonized the inhibitory effect on cumulative food intake (0–6 h) induced by spexin. Spexin significantly reduced the mRNA level of *Npy* mRNA, not *Agrp*, *Pomc*, *Cart*, *Crh*, *Orexin*, or *Mch*, in the hypothalamus. Spexin (10 nmol/mouse, i.p.) increased the number of c-Fos positive neurons in hypothalamic AHA and SCN, but not in ARC, DMN, LHA, PVN, SON, or VMH. The hypothalamic p-CaMK2 protein expression was upregulated by spexin. This study indicated that acute peripheral injection of spexin inhibited mouse food intake. The anorectic effect may be mediated by GALR3, and inhibiting neuropeptide Y (NPY) via p-CaMK2 and c-Fos in the hypothalamus.

## 1. Introduction

Spexin (SPX), also named neuropeptide Q (NPQ), is a novel endogenous peptide. It was first identified in the human genome through the bioinformatics approach (Markov model) [1]. The human *Spexin* gene was located on chromosome 12, namely C12orf39, consisting of 6 exons and 5 introns [2]. In human, *Spexin* encoded a prepropeptide of 116 amino acids, which generated a mature peptide undergoing process of posttranslational modifications [2]. The sequence of mature spexin containing 14 amino acids (NWTPQAMLYLKGAQ) was conserved across vertebrate species [3]. The gene structure analysis indicated that *spexin* was closely related to *galanin* and *kisspeptin* [4]. A ligand-receptor assay revealed that spexin could activate galanin receptors type 2 (GALR2) and 3 (GALR3) [4].

Spexin gene or protein had a wide distribution in the central nervous system (CNS) and peripheral tissues in several species, including human, rodents, chicken, anole lizard, and several fish species [3]. In rat, the spexin protein was observed in stomach fundus, epithelium of small intestine, submucosal layer of esophagus, and hepatocytes [5]. Rat *spexin* gene was found in various tissues including the brain, hypothalamus, esophagus, liver, kidney, thyroid, and ovary [5]. The human *spexin* gene and spexin protein were observed in the stomach, small intestine, pancreatic islets, lung, kidney, and liver [6]. The extensive distribution indicated a crucial role of spexin in biological functions.

Recent studies demonstrated that spexin was involved in multiple biological and pathological roles [7]. It was reported that spexin had effects on regulating bile acid synthesis and metabolism [8], glucose metabolism, and ameliorated insulin resistance [9, 10]. Spexin promoted rat stomach muscle contraction in the explant assay [1] and enhanced mouse gastrointestinal motility *in vivo* [11]. The concentration of serum spexin in obese people was lower than that of the normal weight person in both adult and adolescent [12, 13]. In goldfish, spexin was showed to be involved in reproduction and endocrine and reduced the release of luteinizing hormone *in vivo* and *in vitro* studies [14]. In addition, spexin had a regulatory effect on cardiovascular function [15], nociception [15, 16], and anxiety [17].

High quantity of spexin immunopositive neurons were found in rat hypothalamic paraventricular and supraoptic nuclei [5], suggesting a potential role of spexin in appetite regulation. Wong et al. found that intraperitoneal (i.p.) and intracerebroventricular (i.c.v.) treatment with spexin decreased food intake in goldfish [18]. Zheng et al. found that *spx*^−/−^ mutant zebrafish increased the amount of food intake compared with the wild type [19]. Nevertheless, the role of spexin in feeding regulation in mammalian animal is still extremely poorly studied. The present study was designed to investigate the mechanism and effect of spexin on food intake in mice.

## 2. Materials and Methods

### 2.1. Animals and Chemicals

Male C57BL/6 mice (aged 6–8 weeks) were supplied by the Vital River Company (License No. SCXK (Jing) 2012-0001, Beijing, China). The animals were housed in an environmentally controlled room (22 ± 1°C temperature, 50–60% relative humidity, and 12 h/12 h light/dark cycle) with 5-6 mice per cage and free access to water and food. Mice were allowed to adapt to this environment for at least 7 days before the start of the study. The high-fat diet (HFD) (D12492, 60% fat, 20% protein, 20% carbohydrate, and research diets) was obtained from Opensource Animal Diets (Changzhou) Co., Ltd. (Changzhou, China). The study was approved by the Committee of Medical Ethics and Welfare for Experimental Animals, Henan University School of Medicine (No. HUSOM2019-030).

The peptide spexin was supplied by GL Biochem (Shanghai) Ltd. (Shanghai, China). M871 (GALANIN-(2–13)-GLU-HIS-(PRO)3-(ALA-LEU)2-ALA-AMIDE) was purchased from Abcam Inc. (Burlingame, CA). SNAP 37889 (1-phenyl-3-((3-(trifluoromethyl)phenyl)imino)-1H-indol-2-one) was provided by MedChemExpress (Princeton, NJ). All the administered chemicals were dissolved in sterile normal saline.

### 2.2. Assessment of Food Intake

The mice were freely fed with standard chow diet food or given a deprivation of food for 12 h prior to drugs treatment. Animals were injected with an intraperitoneal (i.p.) infusion of normal saline (NS) or drugs at the onset of the dark cycle (19 : 00) or light cycle (07 : 00) in a volume of 100 *μ*L. Thereafter, the amount of standard diet or high-fat diet consumed by the animals was assessed at 1, 2, 4, 6, and 24 h after i.p. injection, respectively. During this process, the mice were ad libitum accessed water. In order to minimize the stress to the mice during the evaluation, they were individually housed for 2 days before i.p. treatment.

### 2.3. Reverse Transcription and Real Time qPCR

Total RNA was extracted from mouse hypothalamic tissues using TRIzol reagent (Invitrogen, Carlsbad, CA) according to the manufacturer's instructions. The amount and quality of the purified RNA was determined by a NanoDrop 2000 UV-Vis Spectrophotometer (Thermo Scientific, Wilmington, DE, USA). Total RNA (0.5 *μ*g) was reversely transcribed into cDNA using a High-Capacity cDNA Reverse Transcription kit (Applied Biosystems, Foster City, CA, USA). The expression of targeted genes, including neuropeptide Y (*Npy*), agouti gene-related protein (*Agrp*), proopiomelanocortin (*Pomc*), cocaine- and amphetamine-regulated transcript peptide (*Cart*), corticotropin releasing hormone (*Crh*), *Orexin*, and melanin-concentrating hormone (*Mch*), were detected by quantitative real-time PCR (ABI 7500HT detection system) using the SYBR Green Master Mix (Applied Biosystems, USA). Amplification of mRNA was performed with one cycle 95°C for 3 min followed by 40 cycles of 95°C for 30 s and 62°C for 40 s. The melting curves analysis was performed to conform the specificity of PCR reactions. 36B4 was used as an internal control gene. The PCR primers and products sizes of each gene are showed in Table 1. The relative expression level of each target gene was calculated by the 2^–ΔΔCt^ method [20].

### 2.4. Western Blot

Hypothalamus tissues were lysed in 1 × lysis buffer containing 1% EDTA, 1% Halt Protease, and Phosphatase Inhibitor Cocktail (Thermo Scientific, USA). After centrifugation at 15000 g for 10 min at 4°C, the supernatant was collected and the protein concentration was estimated by the bicinchoninic acid protein assay (Beyotime, China). Equal amounts of protein were electrotransferred onto a PVDF membrane by a semidry blotting system (BIO-RAD, USA), following the separation on a 10% sodium dodecyl sulfate-polyacrylamide gel electrophoresis (SDS-PAGE). Then, the blots were blocked with 5% milk in Tris-buffered saline with 0.1% Tween (TBS-T) for 1 hour at room temperature. Subsequently, they were incubated overnight at 4°C with primary antibodies GAPDH (1 : 1000, AF0006, Beyotime, China), CaMK2 (1 : 1000, ab52476, Abcam, UK), ERK1/2 (1 : 1000, ab184699, Abcam, UK), phospho-CaMK2 (1 : 1000, ab32678, Abcam, China), and phospho-ERK1/2 (1 : 1000, ab201015, Abcam, UK). Thereafter, blots were washed three times with TBS-T and incubated with horseradish peroxidase conjugated secondary antibody (1 : 2000, E-AB-1003, Elabscience, China) for 1 h at room temperature. The bands on the membrane were visualized with the Thermo Scientific enhanced chemiluminescence detection system. The GAPDH was used as a house keeping protein, and the band intensity was quantified by ImageJ analysis software.

### 2.5. Immunohistochemistry

Four hours after i.p. injection with spexin, mice were euthanized with an injection of pentobarbital sodium pentobarbital sodium (100 mg/kg, i.p.). Mice were transcardially perfused with ice-cold phosphate-buffered saline, followed by fresh 4% paraformaldehyde solution. The completed brains were quickly removed, postfixed in the same fixative for 24 hours at 4°C. After fixation, brains were dehydrated and embedded in paraffin according to standard procedure. Samples were subsequently sectioned using a vibratome (Leica Biosystems, Nussloch, Germany) in the coronal plane into 5 *μ*m thick. c-Fos immunohistochemistry was performed as previous described [16]. Sections were processed with 10% normal goat serum (Proteintech, Wuhan, China) for one hour at room temperature to block nonspecific antibody binding. Thereafter, they were incubated overnight with a primary antibody (rabbit anti-c-Fos antibody, 1 : 100, Abcam Inc., Burlingame, CA, USA). Subsequently, sections were incubated with a secondary antibody (goat anti-rabbit IgG, 1 : 600, Proteintech, Wuhan, China) for 2 hours at room temperature. Then, it was treated with avidin-biotin complex (Corning Inc., Corning, NY) for 2 hours, and the immunostaining was revealed by the diaminobenzidin reaction. The stereotaxic mouse brain atlas was used to define the areas of the hypothalamus [21]. The number of c-Fos immunoreactive cells in the anterior hypothalamic area (AHA), arcuate nucleus (ARC), dorsomedial nucleus (DMN), lateral hypothalamic area (LHA), paraventricular nucleus (PVN), supraoptic nucleus (SON), suprachiasmatic nucleus (SCN), and ventromedial hypothalamus (VMH) were counted under a light microscope (20x) by a double-blind manner.

### 2.6. Glucose Tolerance Test and Insulin Tolerance Test

For the glucose tolerance test, mice fasted overnight (12 hours) were i.p. injected with d-glucose (2 g/kg). Blood samples were collected from the tail veins at 0, 15, 30, 60, and 120 min after injection. Blood glucose was measured using a glucose meter (Accu-Chek, Roche, Switzerland). For the insulin tolerance test, after deprivation of food for 6 hours, mice were i.p. infused with insulin (1 IU/kg, HVGH852, Novo Nordisk, DK). Blood was collected, and glucose was measured at 0, 15, 30, and 60 min by a similar procedure described above for the glucose tolerance test.

### 2.7. Open Field Test

The open field test was performed using the universal spontaneous activity video analysis system (Model no. JLBehv-LM4, Shanghai Jiliang Software Technology Co., Ltd., Shanghai, China). The instrument contained four cages (25 × 25 × 31 cm), each of which was equipped with a video camera above the center. The movement of the animal was tracked and analyzed by the software system. Mice were habituated in the in the test box for 30 min before i.p. injection, and then, they were put back to the boxes individually for recording for one hour.

### 2.8. Experiment Design

To study the influence of spexin on food intake, the mice fasted for 12 h were immediately i.p. injected with spexin (1, 10 nmol/mouse) or NS at the light onset, and the freely feeding mice were i.p. injected with spexin (1, 10 nmol/mouse) or NS at the dark onset. Then, the food intake for standard diet, which the mice had previously received, was determined. At the same time, the freely feeding mice were i.p. injected with spexin (1, 10 nmol/mouse) or NS at the light onset, and the food intake for high-fat diet, which the mice received for the first time, was detected.

The freely feeding mice could reflect the feeding behavior under natural condition, so the freely feeding mice model was selected for next experiment. In order to determine whether GALR2/3 was involved in the anorectic effect induced by spexin, the GALR2 antagonist M871 (10 nmol/mouse) and GALR3 antagonist SNAP37889 (10 nmol/mouse) were coinjected (i.p.) with spexin (10 nmol/mouse), respectively, in a total volume of 100 *μ*L. To further explore the potential mechanism, 4 hours after injection with 10 nmol spexin or NS, the mouse hypothalamus was quickly isolated from the brain, frozen in liquid nitrogen for 20 min, and instantly transferred to −80°C fridge for RT-qPCR and the Western blot analysis. The other mice were transcardially perfused for brain sections for c-Fos immunohistochemistry experiment. The glucose tolerance test, insulin tolerance test, and spontaneous activity test were performed at 4 hours after spexin or NS infusion.

### 2.9. Data Analysis

All values are presented as mean ± standard error of mean (S.E.M.). Data were analyzed using one-way analysis of variance (ANOVA) followed by the post hoc Dunnett's test. Differences between the two groups were compared using the unpaired Student's *t*-test. *p* < 0.05 was considered to be statistically significant.

## 3. Results

### 3.1. Intraperitoneal Injection of Spexin Inhibited Food Intake in Normal Diet Mice

**Figure 1:**
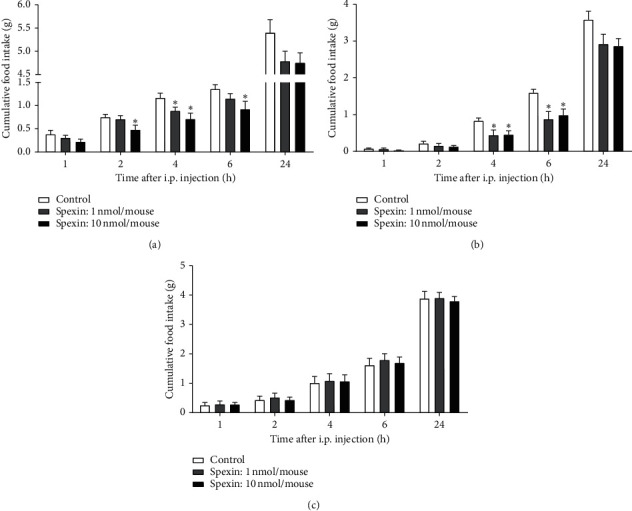
The effect of peripheral injection of spexin (1 and 10 nmol/mouse, i.p.) on food intake in mice (a). The effect of spexin on cumulated food intake in fasted mice during the light period. Spexin or normal saline (NS, control) was injected at the onset of the light cycle (b). The effect of spexin on cumulated food intake in freely feeding mice during the dark period. Spexin or NS was injected at the onset of the dark cycle (c). The effect of spexin on cumulated food intake in high-fat diet mice during the light period. Spexin or NS was injected at the onset of the light cycle. All data are expressed as mean ± S.E.M. *n* = 9−10 per group. ^*∗*^*p* < 0.05 versus the control. One way ANOVA followed by Dunnett's post hoc comparisons was performed.

As shown in Figure 1(a), one-way ANOVA indicated that there was a significant difference between the spexin-treated groups and the control group at 2 h (*F* (2, 26) = 3.502, *p* < 0.05) and 4 h (*F* (2, 26) = 5.091, *p* < 0.05) after i.p. injection in fasted mice during the light period. Post hoc analyses showed that 10 nmol spexin significantly inhibited food intake (normal diet) at 2 h, 4 h, and 6 h after i.p. injection (each *p* < 0.05), and 1 nmol spexin obviously reduced food intake at 4 h after i.p. injection (*p* < 0.05), compared with the control group.

There was a significant difference between the spexin-treated groups and the control group at 4 h (one-way ANOVA, *F* (2, 24) = 4.133, *p* < 0.05) and 6 h (*F* (2, 24) = 5.584, *p* < 0.05) after i.p. injection in freely feeding mice for normal diet during the dark period (Figure 1(b)). Compared with the control group, spexin significantly decreased food intake at 4 h (Dunnett's test, 1 nmol, *p* < 0.05; 10 nmol, *p* < 0.05) and 6 h (1 nmol, *p* < 0.05; 10 nmol, *p* < 0.05) after i.p. injection.

One-way ANOVA demonstrated that there was no difference between the spexin-treated groups and the control group at each time point (1 h, *p* = 0.965; 2 h, *p* = 0.851; 4 h, *p* = 0.971; 6 h, *p* = 0.850; and 24 h, *p* = 0.915) after i.p. injection in freely feeding mice for high-fat diet during the light period (Figure 1(c)).

### 3.2. GALR3 Antagonist SNAP37889, Not GALR2 Antagonist M871, Significantly Blocked the Reduced Food Intake Induced by Spexin in Freely Feeding Mice during the Dark Period

**Figure 2:**
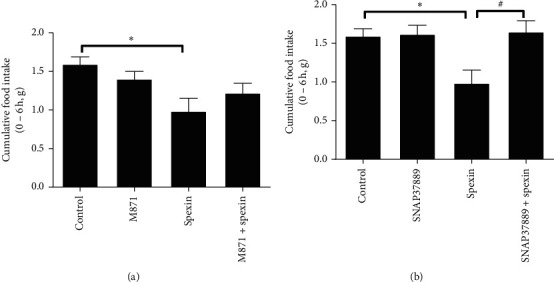
The effect of GALR2 antagonist M871 (a) and GALR3 antagonist SNAP37889 (b) on cumulated food intake (0−6 h) induced by i.p. spexin in freely feeding mice during the dark period. M871 (10 nmol/mouse) and SNAP37889 (10 nmol/mouse) were coadministered (i.p.) with spexin (10 nmol/mouse) at the onset of the dark cycle, respectively. All data are expressed as mean ± S.E.M. *n* = 8−9 per group. ^*∗*^*p* < 0.05 versus the control. ^#^*p* < 0.05 versus the spexin-treated group.

To explore the possible mechanism involved in the inhibition of spexin on feeding intake, the GALR2 antagonist M871 (10 nmol/mouse) and GALR3 antagonist SNAP37889 (10 nmol/mouse) were selected and i.p. coadministered with spexin, respectively. As shown in Figure 2(a), M871 alone had no effect on cumulative food intake in 0–6 h (*p*=0.587, compared with the control). Unexpectedly, coinjection of M871 with spexin could not block the inhibitory effect of spexin on food intake (*p*=0.430, compared with the spexin group), suggesting that GALR2 was not involved in the anorectic effect of spexin.  Figure 2(b) demonstrates that SNAP37889 also did not influence cumulative food intake at 6 h (*p*=0.999, compared with the control). However, it significantly antagonized the inhibition of food intake induced by spexin (*p* < 0.05, compared with the spexin group), indicating that the anorectic effect of spexin was mediated by GALR3.

### 3.3. Spexin Reduced Npy Gene Expression in Hypothalamus of Mice

**Figure 3:**
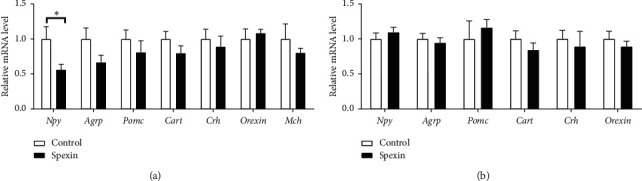
The effect i.p. injection of 10 nmol/mouse spexin on *Npy*, *Agrp*, *Pomc*, *Cart*, *Crh*, *Orexin*, and *Mch* mRNAs levels in the hypothalamus (a) and brainstem (b) in mice. The housekeeping gene 36B4 was used as the internal control. The target genes were detected by RT-qPCR after 10 nmol spexin injection. The unpaired *t*-test was utilized to analyze the difference between the spexin and the control (NS). Data are presented as mean ± S.E.M. n = 6−8 per group. ^*∗*^*p* < 0.05 versus the control.

To determine which appetite kind of regulatory factors was involved in the anorectic effect of spexin, the related genes were detected. Spexin did not influence *Agrp* (*p*=0.126), *Pomc* (*p*=0.373), *Cart* (*p*=0.198), *Crh* (*p*=0.599), *Orexin* (*p*=0.587), or *Mch* (*p*=0.417) gene expression in the hypothalamus of mice, compared with the control group (Figure 3(a)). However, it significantly reduced *Npy* gene expression in the hypothalamus (*p* < 0.05, Figure 3(a)). In the brainstem, spexin did not change the mRNAs level of *Npy* (*p*=0.408), *Agrp* (*p*=0.616), *Pomc* (*p*=0.575), *Cart* (*p*=0.320), *Crh* (*p*=0.663), or *Orexin* (*p*=0.435), compared with the control group (Figure 3(b)).

### 3.4. Spexin Increased the Number of c-Fos Positive Cells in AHA and SCN of Mouse Hypothalamus

**Figure 4:**
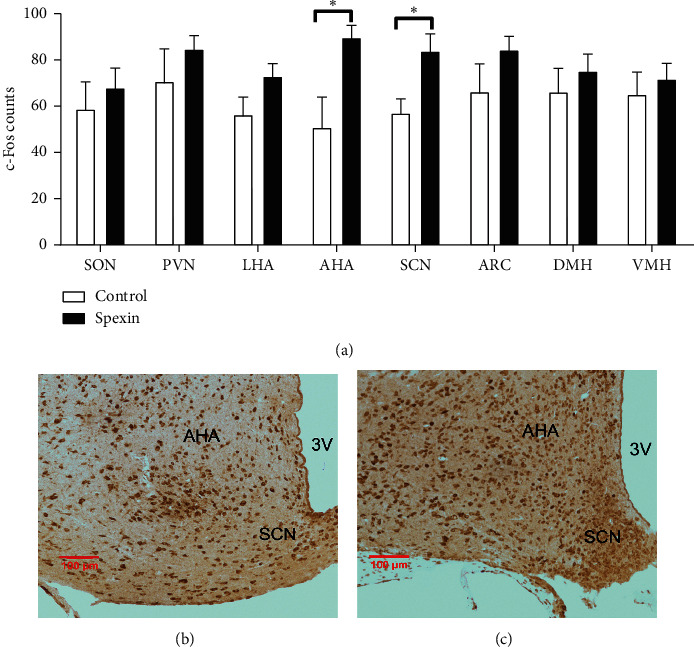
The effect of spexin on c-Fos like immunoreactivity in the hypothalamus of mice (a). Histogram showing the mean number of c-Fos like immunoreactivity neurons in several hypothalamic nuclei of mice after i.p. injection of NS or spexin (10 nmol/mouse). (b, c) Representative brain sections showing c-Fos expression in the AHA and SCN of mice i.p. injected with NS or spexin (10 nmol/mouse). Scale bars are equivalent to 100 *μ*m. Data are showed as mean ± S.E.M. n = 5 per group. ^*∗*^*p* < 0.05 versus the control. 3V, third ventricle.

To investigate the hypothalamic areas that may be involved in the inhibitory effect of spexin on food intake, the number of c-Fos such as immunoreactivity neurons were compared within each subdivision in the mouse hypothalamus. Spexin significantly reduced the number of c-Fos positive cells in AHA and SCN (each *p* < 0.05), but not in ARC (*p*=0.214), DMN (*p*=0.495), LHA (*p*=0.116), PVN (*p*=0.390), SON (*p*=0.543), or VMH (*p*=0.593), compared with the saline control (Figure 4(a)). Figures 4(b) and 4(c) show the examples of c-Fos like immunoreactivity neurons in AHA and SCN of the hypothalamus in mice.

### 3.5. Spexin Upregulated p-CamK2, Not p-ERK1/2, Protein Expression in Mouse Hypothalamus

**Figure 5:**
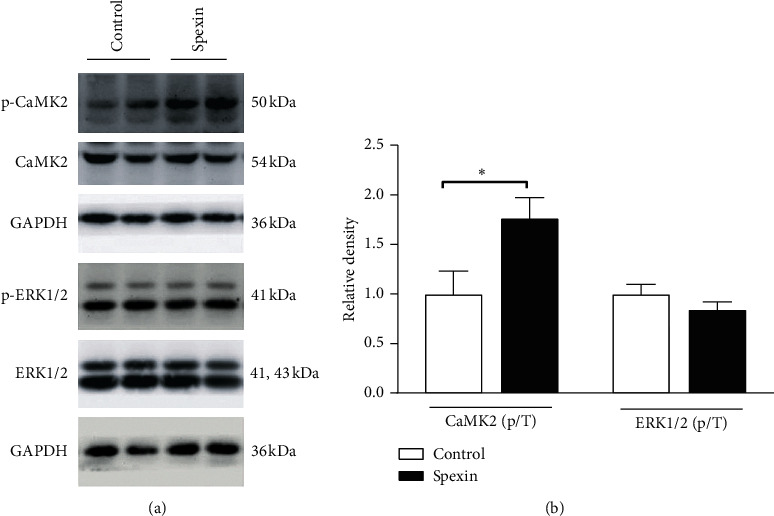
The effect of spexin on p-ERK1/2 and p-CamK2 protein levels in mouse hypothalamus. Representative immunoblot (a) and densitometric determination (b) of p-ERK1/2 and p-CamK2 protein after i.p. injection with NS or spexin (10 nmol/mouse). Data are shown as mean ± S.E.M. *n* = 5−6 per group. ^*∗*^*p* < 0.05 the versus control.

The Western blot analysis indicated that the p-CaMK2 protein level was significantly increased, compared with the control group (*p* < 0.05, Figure 5(a)). In contrast, the protein expression level of p-ERK1/2 did not change markedly, compared with the control group (*p*=0.23, Figure 5(b)).

### 3.6. Spexin Had No Influence on Glucose Tolerance, Insulin Sensitivity, or Spontaneous Activity

During the glucose tolerance test, the spexin-treated group had a slight lower level of blood glucose at 30 min and 60 min after glucose injection, compared with the control group. However, they all did not reach a statistically different level at each time point (15 min, *p*=0.790; 30 min, *p*=0.232; 60 min, *p*=0.136; 120 min, *p*=0.557, supplementary Figure 1(a)), and the AUC had no significant difference between the spexin-treated group and the control (*p*=0.26, supplementary Figure 1(b)). During the insulin tolerance test, there was no significant difference between the spexin group and the control group at 15 min (*p*=0.604), 30 min (*p*=0.459), or 60 min (*p*=0.625) after insulin treatment, respectively (supplementary Figure 1(c)), and it had no significant difference between the AUC of the spexin group and the control group (*p*=0.80, supplementary Figure 1(d)).

One-way ANOVA indicated that there was no difference between the spexin-injected groups (1, 10 nmol/mouse) and the control group for the spontaneous activity (*F* (2, 27) = 0.341, *p*=0.714, supplementary Figure 2(a)), total distance travelled (*F* (2, 27) = 0.746, *p*=0.484, supplementary Figure 2(b)), and average velocity (*F* (2, 27) = 0.746, *p*=0.484, supplementary Figure 2(c)).

## 4. Discussion

Our results indicated that acute peripheral injection of spexin inhibited food intake for standard diet in freely feeding mice during the dark period and fasted mice during the light period. However, spexin had no influence on food intake of high-fat diet in mice during the light period. The anoretic effect of spexin was supported by the anatomical evidence that spexin immunoreactivity had been found in paraventricular nucleus, supraoptic nucleus, and other hypothalamic nuclei in the hypothalamus of rodents [5]. To investigate the acute effects of drugs on satiation or satiety in rodents, nocturnal feeding behavior was regularly assessed because mice eat their largest meals soon after “lights off” [22]. In laboratory mice, fasting triggers a hyperphagic response during the refeeding period, and fasting-induced feeding is often adopted to determine anorexigenic behavior [22]. Our results demonstrated the consistently anorectic effect of spexin under the dark period and fasting condition in mice. In fasted flatfish, the *spexin* gene expression in the hypothalamus was increased compared with the freely feeing group [23]. Wong et al. found that intraperitoneal (1–10000 ng/g) and intracerebroventricular (1–100 ng/g) spexin suppressed 2 h food intake in goldfish [18], which is in accordance with our finding in this study.

Spexin was coevolved with the galanin/kisspeptin family, and it was confirmed that spexin could activate GALR2/3 receptors [4]. GALR2 antagonist M871 and GALR3 antagonist SNAP37889 had been used to the role of spexin in gastrointestinal motility [11], inflammatory pain [16], bile acid synthesis [8], lipolysis [24], and *in vivo* or *in vitro* studies. Our result demonstrated that SNAP37889 (*p* < 0.05), not M871 (*p*=0.430), significantly antagonized the inhibitory effect of spexin on food intake, suggesting that GALR3, not GALR2, was involved in the anorectic effect of spexin.

The hypothalamus was considered as the crucial center for modulating energy intake and expenditure in mammals. Several nucleus of the hypothalamus are in response to peripheral signals and induces secretion of orexigenic and anorexigenic neuropeptides, which are implicated in modulation of food intake and energy homeostasis [25, 26]. The genes of neuropeptides related to appetite regulation were determined. The result demonstrated that spexin obviously suppressed hypothalamic *Npy* mRNA expression, while it did not influence *Agrp*, *Pomc*, *Cart*, *Crh*, *Orexin*, or *Mch* mRNAs. In the CNS, the brainstem also played a role in energy homeostasis [27]. Unexpectedly, spexin did not significantly change *Npy*, *Agrp*, *Pomc*, *Cart*, *Crh*, or *Orexin* mRNAs in the brainstem. NPY, an orexigenic neuropeptide, is one of the strongest orexigenic signals in the hypothalamus [28]. These results suggested that the anorectic effect of spexin was related to downregulation of *Npy* in the hypothalamus.

c-Fos was identified as a marker of neuronal activation [29]. To explore the hypothalamic sites that may mediate the anorectic effect of spexin, c-Fos immunoreactivity was detected by immunohistochemistry. Our result indicated that i.p. injection of spexin significantly resulted in c-Fos induction in AHA and SCN (each *p* < 0.05), but not in ARC, DMN, LHA, PVN, SON, or VMH, suggesting that the anorectic effect of spexin may be mediated via hypothalamic AHA and SCN. Wu et al. found that c-Fos could regulate NPY expression in mouse dentate gyrus [30]. We could suppose that the decrease of *Npy* mRNA induced by spexin might be due to the c-Fos activity in the hypothalamus. CaMK2 and ERK1/2 are important signal molecules to regulate the nuclear transcription factor c-Fos [31, 32]. Our result indicated that spexin significantly increased the p-CaMK2 protein level in the mouse hypothalamus (*p* < 0.05), while it did not influence the p-ERK1/2 protein level (*p*=0.253). These indicated that p-CaMK2 was involved in the anorectic effect of spexin. In addition, we found that acute i.p. injection of spexin did not acutely influence glucose tolerance, insulin sensitivity, or spontaneous activity in mice.

In summary, we found that acute peripheral injection of spexin (1, 10 nmol/mouse) inhibited food intake in mice. Spexin may act on GALR3, then induce c-Fos expression in AHA and SCN via p-CaMK2, and suppress hypothalamic NPY expression, thereby producing the anorectic effect.

## Figures and Tables

**Table 1:** Primer sequence used for RT-qPCR.

Primers name	Primer sequence	Size (bp)
*Npy*-F	5′- AATCAGTGTCTCAGGGCTGGAT-3′	74
*Npy*-R	5′-CCGCTCTGCGACACTACATC-3′	
*Agrp*-F	5′-CGGAGGTGCTAGATCCACAGA-3′	69
*Agrp*-R	5′-AGGACTCGTGCAGCCTTACAC-3′	
*Pomc*-F	5′-AAGAGCAGTGACTAAGAGAGGCCA-3′	159
*Pomc*-R	5′-ACATCTATGGAGGTCTGAAGCAGG-3′	
*Cart*-F	5′-GCGCTATGTTGCAGATCGAA-3′	105
*Cart*-R	5′-CGTCACACATGGGGACTTGG-3′	
*Crh*-F	5′-TCAGAGCCCAAGTACGTT-3′	113
*Crh*-R	5′-AGGGACTTCTCTCAGGAT-3′	
*Orexin*-F	5′-GACGGCCTCAGACTTCTTGG-3′	118
*Orexin*-R	5′-GGCCCAGGGAACCTTTGTAG-3′	
*Mch*-F	5′-TCCAATGCACTCTTGTTTGGC-3′	310
*Mch*-R	5′-TGTTTGGAGCCTGTGTTCTTTG-3′	
*36B4*-F	5′-CGACCTGGAAGTCCAACTAC-3′	109
*36B4*-R	5′-ATCTGCTGCATCTGCTTG-3′	

F, forward; R, reverse.

## Data Availability

The data used to support the findings of this study are available from the corresponding author upon request.

## Abbreviations

AHA: Anterior hypothalamic area
ARC: Arcuate nucleus
AUC: Area under the curve
CaMKII: Calmodulin-dependent kinase II
CNS: Central nervous system
DMN: Dorsomedial nucleus
ERK1/2: Extracellular signal-regulated kinase1/2
GALR2: Galanin receptors type 2
GALR3: Galanin receptors type 3
i.c.v.: Intracerebroventricular
i.p.: Intraperitoneal
LHA: Lateral hypothalamic area
NPQ: Neuropeptide Q
NPY: Neuropeptide Y
NS: Normal Saline
PVN: Paraventricular nucleus
SCN: Suprachiasmatic nucleus
SDS-PAGE: Sodium dodecyl sulfate -polyacrylamide gel electrophoresis
SON: Supraoptic nucleus
SPX: Spexin
VMH: Ventromedial hypothalamus.

## Supplementary Materials

Supplementary Materials Supplementary Figure 1: the effect of spexin on glucose tolerance and insulin tolerance. Supplementary Figure 2: the effect of spexin on the spontaneous activity, total distance travelled, and average velocity in mice.
