# Supplementary material for: Peripheral Spexin Inhibited Food Intake in Mice
Source: Int J Endocrinol. 2020 Aug 5;2020:4913785. doi: 10.1155/2020/4913785 (PMC7426757; doi:10.1155/2020/4913785)
Supplement: Supplementary Materials — Supplementary Figure 1: the effect of spexin on glucose tolerance and insulin tolerance. Supplementary Figure 2: the effect of spexin on the spontaneous activity, total distance travelled, and average velocity in mice. [file 4913785.f1.docx]

Supplementary material


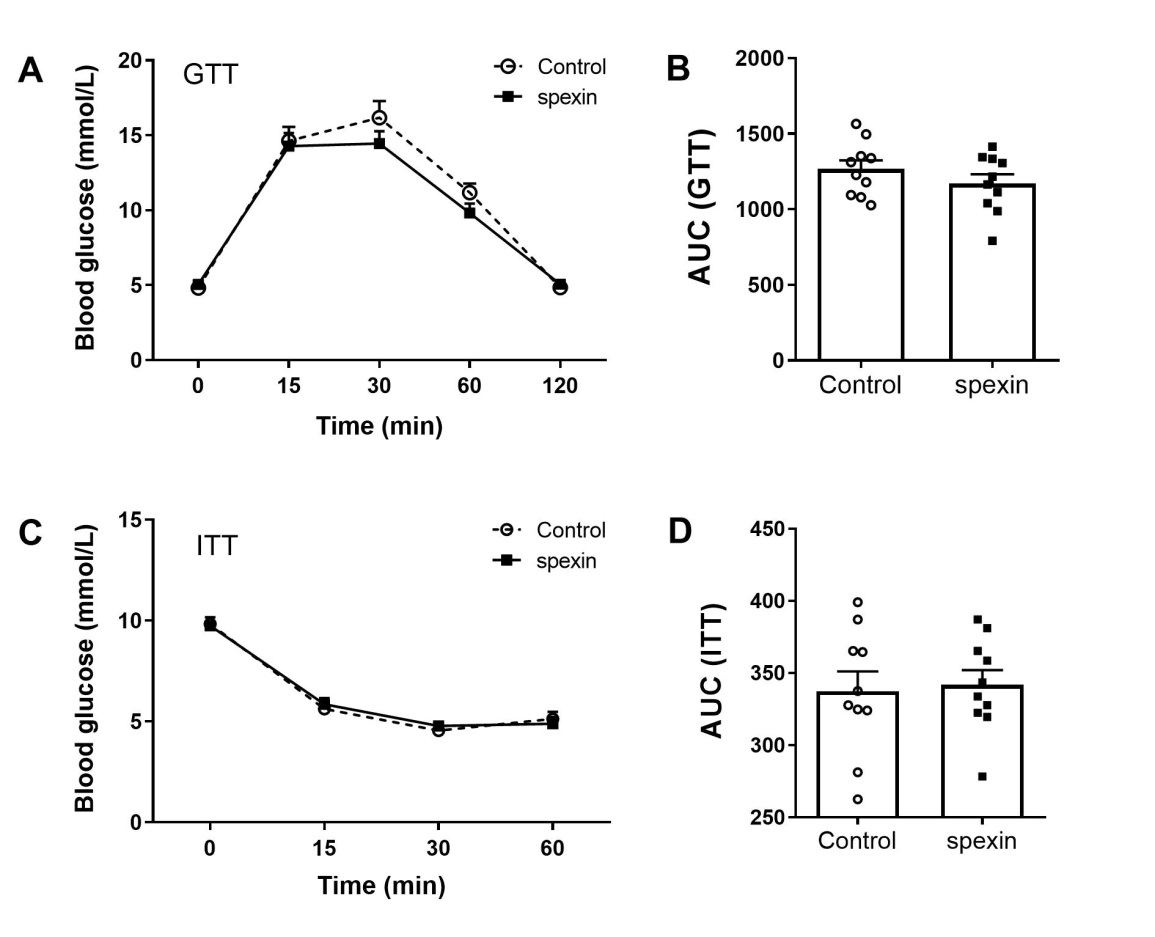


**Supplamentary Fig. 1.** The effect of spexin on glucose tolerance and insulin tolerance. The concentration time curve (A) and area under the concentration time curve (B) during glucose tolerance test (GTT, 2 g/kg glucose, i.p.), and the concentration time curve (C) and area under the concentration-time curve (D) during insulin tolerance test (ITT, 1 IU/kg human insulin, i.p.). Glucose or insulin was injected at 4 h after NS or spexin (10 nmol/mouse) treatment. Data was showed as mean ± S.E.M. n = 10 per group. * p < 0.05, versus control. The difference between spexin and the control (NS) at each time point was performed by unpaired t-test. AUC, area under the curve.


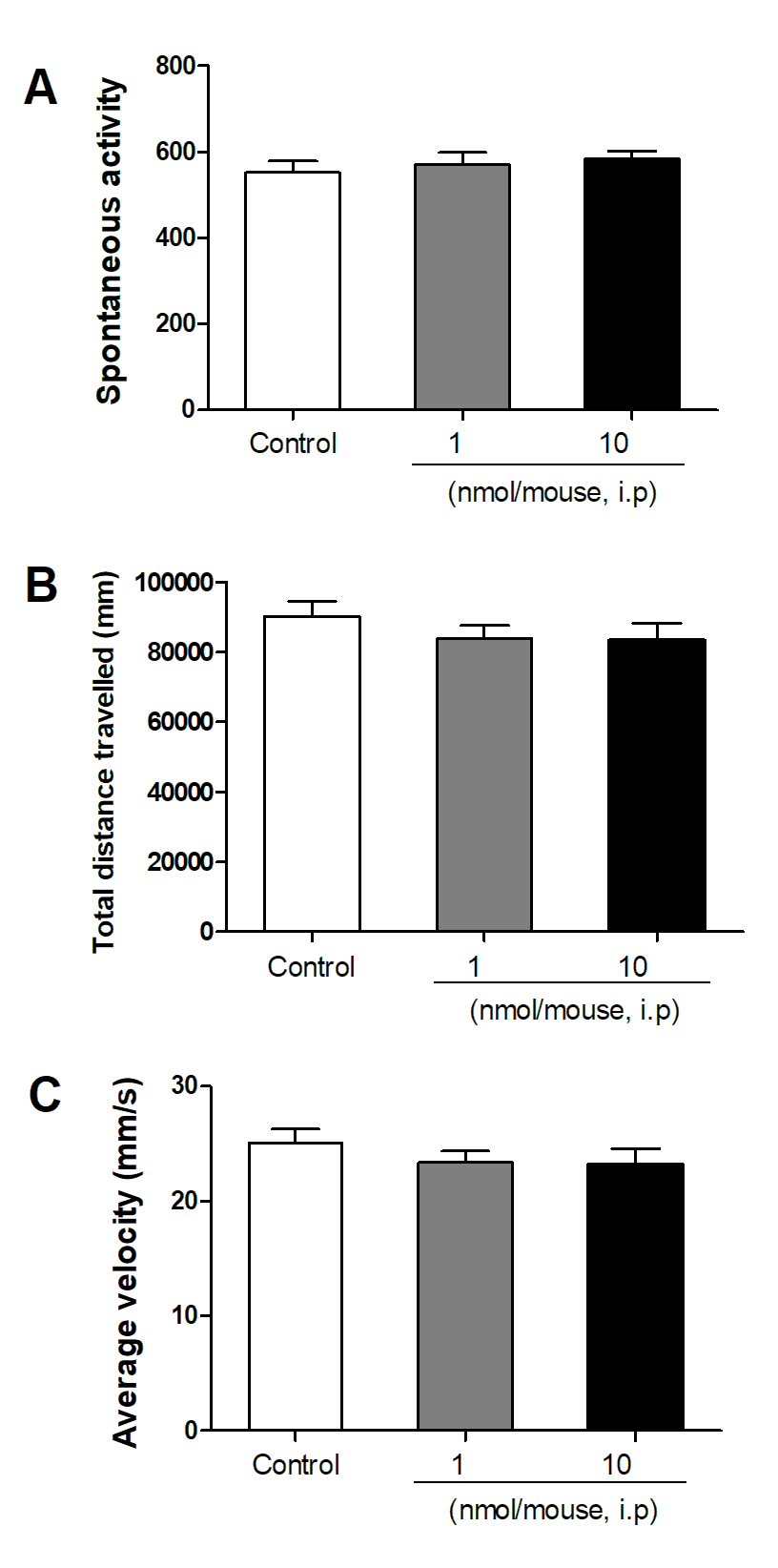


**Supplamentary Fig. 2.** The effect of spexin on spontaneous activity (A), total distance travelled (B), and average velocicity (C) in mice. The activity of mice was recorded for 1 hour after NS or spexin (1, 10 nmol/mouse) injection. Data was showed as mean ± S.E.M. n = 10 per group. ANOVA followed by Dunnett’s test was used for analysis.
